# Hepcidin as a key iron regulator mediates glucotoxicity-induced pancreatic β-cell dysfunction

**DOI:** 10.1530/EC-18-0516

**Published:** 2019-01-21

**Authors:** Tingting Shu, Zhigang Lv, Yuchun Xie, Junming Tang, Xuhua Mao

**Affiliations:** 1 Department of Central Laboratory, Jiangsu Province Official Hospital, Nanjing, Jiangsu, China; 2 Department of Clinical Laboratory, Yixing People Hospital, Affiliated Jiangsu University, Yixing, Wuxi, Jiangsu, China

**Keywords:** T2DM, hepcidin, iron overload, pancreatic dysfunction

## Abstract

It has been well established that glucotoxicity induces pancreatic β-cells dysfunction; however, the precise mechanism remains unclear. Our previous studies demonstrated that high glucose concentrations are associated with decreased hepcidin expression, which inhibits insulin synthesis. In this study, we focused on the role of low hepcidin level-induced increased iron deposition in β-cells and the relationship between abnormal iron metabolism and β-cell dysfunction. Decreased hepcidin expression increased iron absorption by upregulating transferrin receptor 1 (TfR1) and divalent metal transporter 1 (DMT1) expression, resulting in iron accumulation within cells. Prussia blue stain and calcein-AM assays revealed greater iron accumulation in the cytoplasm of pancreatic tissue isolated from *db/db* mice, cultured islets and Min6 cells in response to high glucose stimulation. Increased cytosolic iron deposition was associated with greater Fe^2+^ influx into the mitochondria, which depolarized the mitochondria membrane potential, inhibited ATP synthesis, generated excessive ROS and induced oxidative stress. The toxic effect of excessive iron on mitochondrial function eventually resulted in impaired insulin secretion. The restricted iron content in *db/db* mice via reduced iron intake or accelerated iron clearance improved blood glucose levels with decreased fasting blood glucose (FBG), fasting blood insulin (FIns), HbA1c level, as well as improved intraperitoneal glucose tolerance test (IPGTT) results. Thus, our study may reveal the mechanism involved in the role of hepcidin in the glucotoxcity impaired pancreatic β cell function pathway.

## Introduction

Hepcidin is synthesized and secreted primarily in the liver and is a key regulator in iron metabolism (1, 2). In 2008, Kulaksiz *et al.* first reported that hepcidin was expressed in human and rat islet tissues and only existed in insulin-secreting β-cells (3), and then other studies showed low concentration of glucose could stimulate hepcidin secretion in pancreatic beta cell line (4, 5). Subsequently, the correlation between hepcidin and type 2 diabetes (T2DM) has gained increased attention. In addition, several reviews have indicated that hepcidin is an independent risk factor for the onset of T2DM (6, 7, 8). Indeed, the serum hepcidin levels in T2DM patients has been found to be significantly lower than those in healthy individuals (7, 9, 10). However, the mechanism by which hepcidin mediates T2DM pathogenesis remains unclear. Recent reports attribute the probable mechanism to the induction of peripheral tissue insulin resistance (11) through inflammatory response, oxidative stress (12) and mitochondrial dysfunction pathways (13), which affect glucose metabolism in peripheral tissues (5). To date, there have been few reports describing the role of hepcidin in pancreatic β-cells. Our previous results confirmed that the level of hepcidin was decreased under conditions of high glucose stimulation and had a disrupted effect on insulin secretion (14). In addition, the iron status induced by decreased hepcidin expression and its possible toxic effect on β-cell function must be further evaluated and explored.

The lower level of hepcidin could cause iron overload by preventing iron exportation or increase iron intake (15, 16). The deposition of iron in the cytosol is pumped into mitochondria via the ion transporter mitochondrial substrate carrier family protein (Mcfu) (17, 18). As a divalent positively charged ion, Fe^2+^ depolarizes the mitochondria membrane potential, resulting in a disruption of the electron transport chain (ETC), (19) which influences the energy supply required for insulin secretion (20). Moreover, mitochondrial function becomes impaired, which induces endoplasmic reticulum stress response (ER stress), leading to β-cell apoptosis (21, 22). In the situation described earlier, we believe that the iron overload in β-cells induced by low hepcidin levels plays an important role in the process of glucotoxicity-mediated depression of β-cell function.

In this study, we aimed to clarify the iron overload status in the cytosol and mitochondria using the Min6 cell line, pancreatic islets and *db/db* mice, as well as discuss the probable mechanism by which iron toxicity influences mitochondrial function. To this end, we used *db/db* mice to study the effect of restricting iron content on blood glucose levels by decreasing iron intake or accelerating iron clearance.

## Materials and methods

### Cell culture

The mouse pancreatic β-cell line, Min6 (passage 15–28 was kindly providing by department of islet β-cell function laboratory, Jiangsu Province Official Hospital), was cultured in Dulbecco’s modified Eagles medium (Invitrogen) containing 25 mM glucose and supplemented with 15% fetal bovine serum (Invitrogen). The media was supplemented with 100 μg/mL streptomycin, 100 U/mL penicillin and 50 μmol/L β-mercaptoethanol. The cells were maintained at 37°C in a humidified incubator under 5% CO_2_/95% air.

### Virus construction and gene infected

The mouse hepcidin-expressing plasmid was constructed by inserting the full-length coding region of hepcidin (ID:84506) into pCDNA 3.0 vector, and then cut from pCDNA 3.0 ligated into Ad-track vector (Ad-hepcidin) and sequenced to confirm.

For gene transfer, adenovirus generation, amplification and titration were performed. Viral particles were purified using the Adenovirus Purification Kit (Cell Biolabs, San Diego, CA, USA). Min6 cells were infected with adenovirus at a multiplicity of infection of 50 at 37°C and, 2 h after infection, the cells were cultured in fresh medium for another 18 h before treating with glucose (Sigma-Aldrich) treatment. Use Ad-Gfp virus as control.

### Pancreatic islet isolation

All animal studies were performed according to guidelines established by the Research Animal Care Committee of Nanjing Medical University. Animals used for islet isolation (8-week-old C57BL/6 mice) were purchased from the National Resource Center. Islets were isolated and cultured as previously described (23). Insulin immunofluorescence was performed to identify the islet cells (Supplementary Fig. 1, see section on supplementary data given at the end of this article).

### GSIS assay

The GSIS assay was performed as previously described (14) – isolated mouse islets and Min6 cells were transferred into 48-well plates (10 islets/well; 10^4^ cells/well) and treated with different concentrations of glucose. Insulin content was assessed using a commercial ELISA kit (ALPCO Diagnostics) (24) in accordance with the manufacturer’s instructions.

### Hepcidin and ferritin content analysis

The hepcidin content of both the islets and culture supernatants was determined using a commercial ELISA kit purchased from DRG Instruments (GmbH, Marburg, Germany) according to the manufacturer’s protocol. The *db/db* mice and their littermate control mice’ blood ferritin levels were measured using a commercial ELISA kit purchased from Monobind (Lake Forest, CA, USA) according to the manufacturer’s instructions.

### RNA extraction, reverse transcription and qRT-PCR

Total RNA was extracted using TRIzol reagent (Invitrogen) according to the manufacturer’s protocol. Reverse transcription was performed using One-Step RT-PCR System (Invitrogen). SYBR Green Real-time PCR Master Mix (Invitrogen) and Light Cycler 480 II Sequence Detection System (Roche) were used for qRT-PCR, and mRNA levels were normalized to β-actin. The sequences of the primers used for qRT-PCR are listed in Supplementary Table 1. 

### Western blotting

Cells were immediately washed with ice-cold phosphate buffered saline (PBS) and lysed with lysis buffer containing 50 mM Tris–HCl (pH 8.0), 150 mM NaCl, 0.02% sodium azide, 0.1% SDS, 1 μg/mL aprotinin, 1% NP-40, 1% deoxycholic acid sodium salt and 100 μg/mL PMSF. Cell debris was removed by centrifugation (12,000 ***g*** at 4°C for 20 min). The protein concentration was determined using a DC Protein Assay Kit (Bio-Rad), and the protein samples were separated by SDS-PAGE, transferred to Immune-Blot PVDF membranes (Bio-Rad) and incubated at 4°C overnight with rabbit anti-DMT1 (divalent metal transporter 1); rabbit anti-TfR1 (transferrin receptor 1) (Santa Cruz). The membranes were then incubated at room temperature with rabbit anti-β-action antibodies (Santa Cruz) for 1 h and analyzed using the ECL (enhanced chemiluminescence) purchased from Sigma-Aldrich.

### Fluorescence *in situ* hybridization (FISH)

Pancreatic tissues isolated from control and *db/db* mice were fixed in 40 mg/mL in paraformaldehyde and frozen in OCT compound (Sakura, Coronado, CA, USA). For FISH, the probe mixture was dissolved in hybridization buffer (10% dextran sulfate, 2 mM vanadyl-ribonucleoside complex, 0.02% BSA, 2 × SSC, and 10% formamide) and added to the tissue sections for an overnight incubation at 37°C. The probe sequences are listed in Supplementary Table 2. After incubating with the secondary antibodies, images were obtained and analyzed using IX73 fluorescence microscope (Olympus).

### Cytosolic chelatable iron assay

To visualize cytosol iron mobilization of Min6 cells, the cells were grown in 96-well plates and co-loaded with diluted calcein-AM purchased from Life Technologies. The total volume of the culture medium per well for a 96-well plate was 200 µL, which included 100 µL of the initial culture medium, 50 µL of the test compound and 50 µL calcein-AM; all the wells contained 0.1% DMSO. Both fluorescent and phase-contrast images were taken using a fluorescence microscope (Olympus) at the indicated time intervals. Compounds that autofluoresced were excluded. Quenching of calcein-AM fluorescence signifies an increase in cytosolic chelatable Fe^2+^.

### Prussian blue staining

Pancreatic tissues and isolated islet cells from control and *db/db* mice were stained with Perls’ reagent (Sigma) to identify the presence of iron particles. The sections and cells were incubated with Perl’s reagent with 1:1 mixture of 2% potassium ferrocyanide and 1% hydrochloric acid for 30 min at room temperature. The slides were counterstained using nuclear fast red. Prussian blue-positive cells were examined using an Olympus light microscope and photographed.

### ROS determination

Intracellular ROS were measured by flow cytometry using 2, 7-dichlorofluorescein diacetate (DCFH-DA) (BD, Franklin Lakes, NJ, USA) as a probe. After treating the cells with different concentrations of glucose, the cells were washed twice with PBS and co-incubated with serum-free RPMI 1640 containing 10 μM DCFH-DA for 30 min at 37°C in the dark and washed twice with PBS. ROS were measured using Canton II flow cytometer (BD), at 488 nm excitation and 525 nm emission. The data were recorded using Diva software (BD).

### Determination of mitochondrial membrane potential (Δψm)

We followed the methods of Qiao* et al.* 2015 (25). The Δψm in Min6 cells was measured using a MitoScreen (JC-1) kit (BD). The cells were harvested and incubated with JC-1 at 37°C for 15−20 min, after which the staining solution was removed, washed and re-suspended in PBS. The samples were then analyzed with a Canton II flow cytometer (BD). The loss of Δψm was reflected by increased green fluorescence from the JC-1 monomers, as well as a loss of red fluorescence from the JC-1 aggregates.

### Determination of adenosine 5′-triphosphate (ATP) release

ATP release from the cultured cell lines was measured using a commercially available rLuciferase/Luciferin (rL/L) reagent assay (Promega Enliten). Briefly, the samples were neutralized to pH 7.4 with 10 μL 4M Tris and were aliquoted to a new tube with 90 μL ATP-free water. Luciferase reagent was added 1 s before measurement in the 20/20n Luminometer Turner BioSystems (Sunnyvale, CA, USA). An ATP standard curve was constructed, and all samples were measured in duplicate. To ensure a low background, a ‘blank’ containing only rL/L reagent and HBSS was analyzed. ATP concentrations were determined by comparison to a standard curve.

### Animals, treatment and blood parameter determination

Male, four-week-old *db/db* mice and their littermate controls were purchased from Shanghai Laboratory Animal Centre (Shanghai, China). All mice were housed in cages and maintained on a 12 h light/darkness cycle with free access to food and water. The mice were raised for 7 weeks, during which time they were fed a normal chow diet (iron content: 350–600 mg/kg), low iron chow diet (iron content: 35 mg/kg) or a normal chow diet plus iron chelator. The mouse chow were purchased from Harlan Teklad (Madison, WI, USA) (26). Iron chelator referred to as FBS0701 was purchased from FerroKin BioSciences (San Carlos, CA, USA), a magnesium salt of (S)-3´-(OH)-DADFT (27). Drug was dosed at 10 mg/kg, provided once a day. The *db/db* mice were divided into four groups (10 mice/group): (1) *db/db*; (2) *db/db* + low iron diet; (3) *db*/*db* + iron chelator and (4) littermate control mice. Body weight and fasting blood glucose (FBG) were monitored weekly, with FBG levels determined 4 h after removing food. Fasting blood insulin (FIns), HbA1c% and an intraperitoneal glucose tolerance test (IPGTT) were monitored at 4 and 10 weeks. HbA1c% was estimated via liquid chromatography (Sysmex, Tokyo, Japan). IPGTT was performed in the morning with an intraperitoneal injection of 1 g/kg glucose after 12-h fasting. Blood glucose levels were measured at 0, 15, 30, 60 and 120 min and the area under the curve (AUC) for blood glucose was analyzed with Graphpad Prism 6. All animal experimental procedures were performed in accordance with the guidelines established by the Research Animal Care Committee of Nanjing Medical University.

### Statistical analysis

Results are presented as means ± standard error of the mean (s.e.m.). Comparisons between pairs of groups were performed using Student’s *t*-test or using ANOVA for comparisons of multiple groups with SPSS 20.0 software. *P* values <0.05 were considered to indicate statistical significance.

## Results

### Hyperglycemia inhibits hepcidin expression in *db/db* mice, cultured mouse islets and Min6 cells

**Figure 1:**
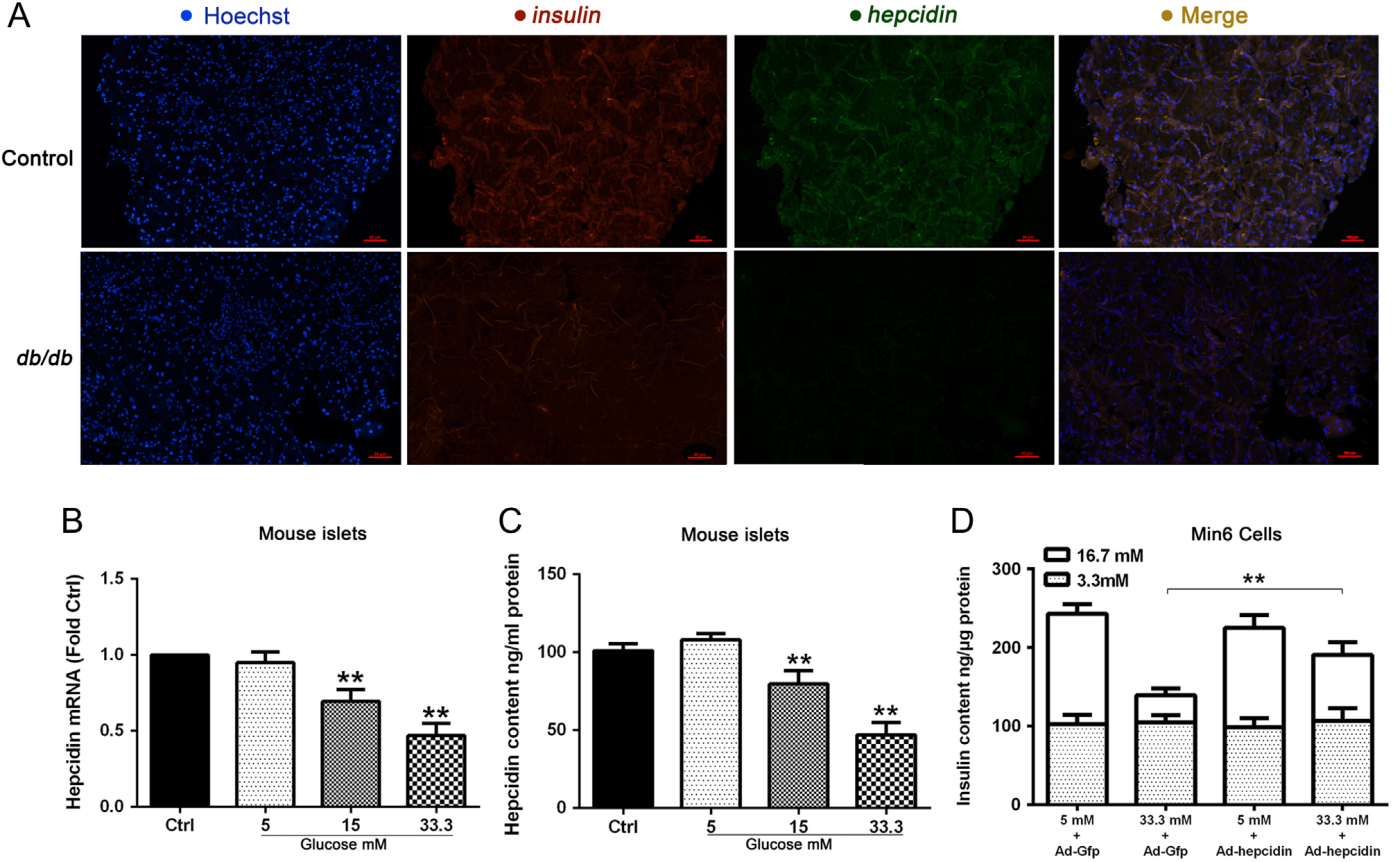
The level of *hepcidin* expression in control mice and *db/db* mice pancreatic tissue (A). Relative mRNA levels of hepcidin were quantified using qRT-PCR analysis with *β-actin* as an internal control in isolated islets with different concentrations of glucose treatment for 48 h. ** indicate *P* < 0.01 compared with the control group (B). Hepcidin content was analyzed in isolated islets using an ELISA method with different concentration of glucose treatment for 48 h. ** indicate *P* < 0.01 compared with the control group (C). GSIS was measured as insulin secretion normalized to the insulin content with an ELISA method in Min6 cell with different concentrations of glucose treatment for 48 h. ** indicate *P* < 0.01 in the Ad-hepcidin-infected group compared with the Ad-Gfp-infected group with 33.3 mM glucose treatment (D). Values are expressed as the means ± s.d. and are representative of three individual experiments.

Hepcidin is expressed in pancreatic β-cells and can be released by secretory granules under glucose stimulation (14). To assess the effect of hyperglycemia on hepcidin expression, we determined the level of hepcidin mRNA expression, protein and secretion content level in *db/db* mouse islets, high glucose cultured mouse islets and Min6 cells. Double immunofluorescent analysis was performed to determine the level of *insulin-1* and *hepcidin* expression. Pancreatic islets of the mouse in the control group strongly expressed *insulin-1* (red) and* hepcidin* (green), whereas in the *db/db* group, both *insulin-1* and *hepcidin* expression were substantially decreased (Fig. 1A). Both the *hepcidin* mRNA level and secretion content decreased in isolated islets following high glucose stimulation (Fig. 1B and C).

To explore whether hyperglycemia impaired GSIS function was related to the low level of hepcidin, we infected an Ad-hepcidin virus to Min6 cells. The *hepcidin* mRNA level was determined to confirm the expression efficiency (Supplementary Fig. 2). The GSIS function was significantly restored compared with Ad-Gfp group (*P* < 0.01) (Fig. 1D).

### Low hepcidin expression induces iron overload in pancreatic β-cells

**Figure 2:**
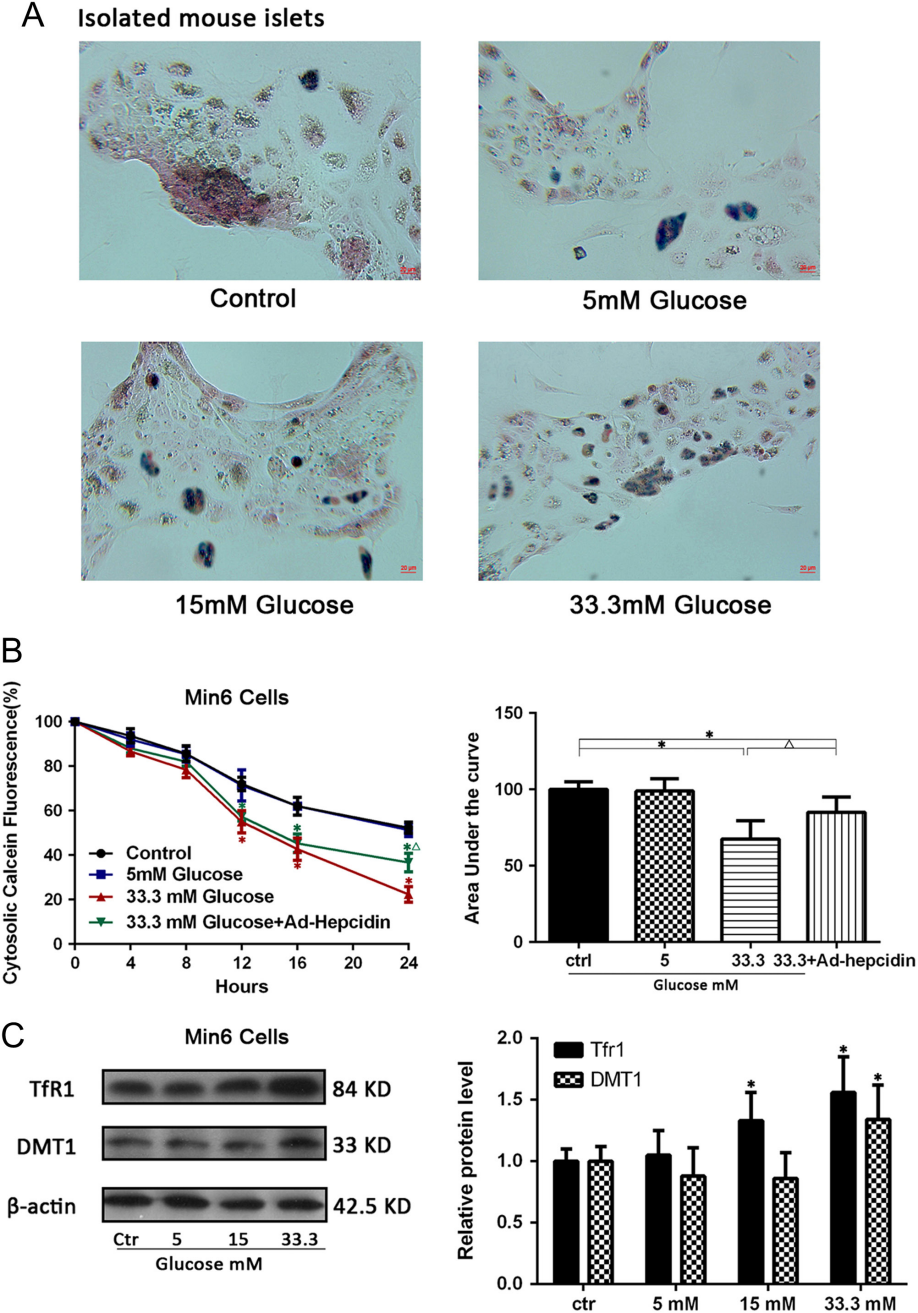
The iron content measured by Prussian blue staining in isolated mouse islets stimulated with different concentrations of glucose for 48 h (A). Cytosolic iron mobilization was measured with calcein-AM fluorescence staining in Min6 cells with different treatments for 48 h. Quenching of calcein-AM fluorescence signifies an increase in cytosolic chelatable Fe^2+^. Fluorescence% was quantified as the area under the curve (AUC) from cytosolic calcein fluorescence%.*indicate *P* < 0.05 compared with the control group; ^△^indicated *P* < 0.05 compared with the 33.3 mM treatment group (B). DMT1 and TfR1 proteins were extracted from Min6 cells with different concentration of glucose treatment for 48 h, analyzed by Western blot; the right panel showed the relative quantification of the normalized DMT1; TfR1 levels to β-actin. Values are expressed as the means ± s.d. and are representative of three individual experiments. *indicate *P* < 0.05 compared with the control group (C).

Hepcidin plays a key role in iron homeostasis (28). Using a Prussia blue stain assay, we assessed the iron content in isolated mouse islets cultured under high glucose conditions. Consistent with our expectations, the iron content increased in a dose-dependent manner with an increase of glucose concentration stimulation as indicated by the blue-stained spots (Fig. 2A). To visualize iron mobilization into the cytosol under hyperglycemia stimulation, Min6 cells were co-loaded with calcein-AM. Quenching of calcein fluorescence signified an increase in the level of cytosolic chelatable iron. The fluorescence intensity was strongly decreased in the 33.3 mM glucose stimulation group but partially recovered with Ad-hepcidin-infected group (Fig. 2B). There was no statistically significant difference in fluorescence intensity between the 33.3 mM group and the 33.3 mM + Ad-Gfp group at each time point (*P* = 0.52). The cytosolic iron overload probably due to iron intake increase via divalent metal transporter (DMT-1) and transferrin receptor 1 (TfR1) protein level increased (Fig. 2C).

### Iron aggregation induces ROS generation which impairs mitochondria function

**Figure 3:**
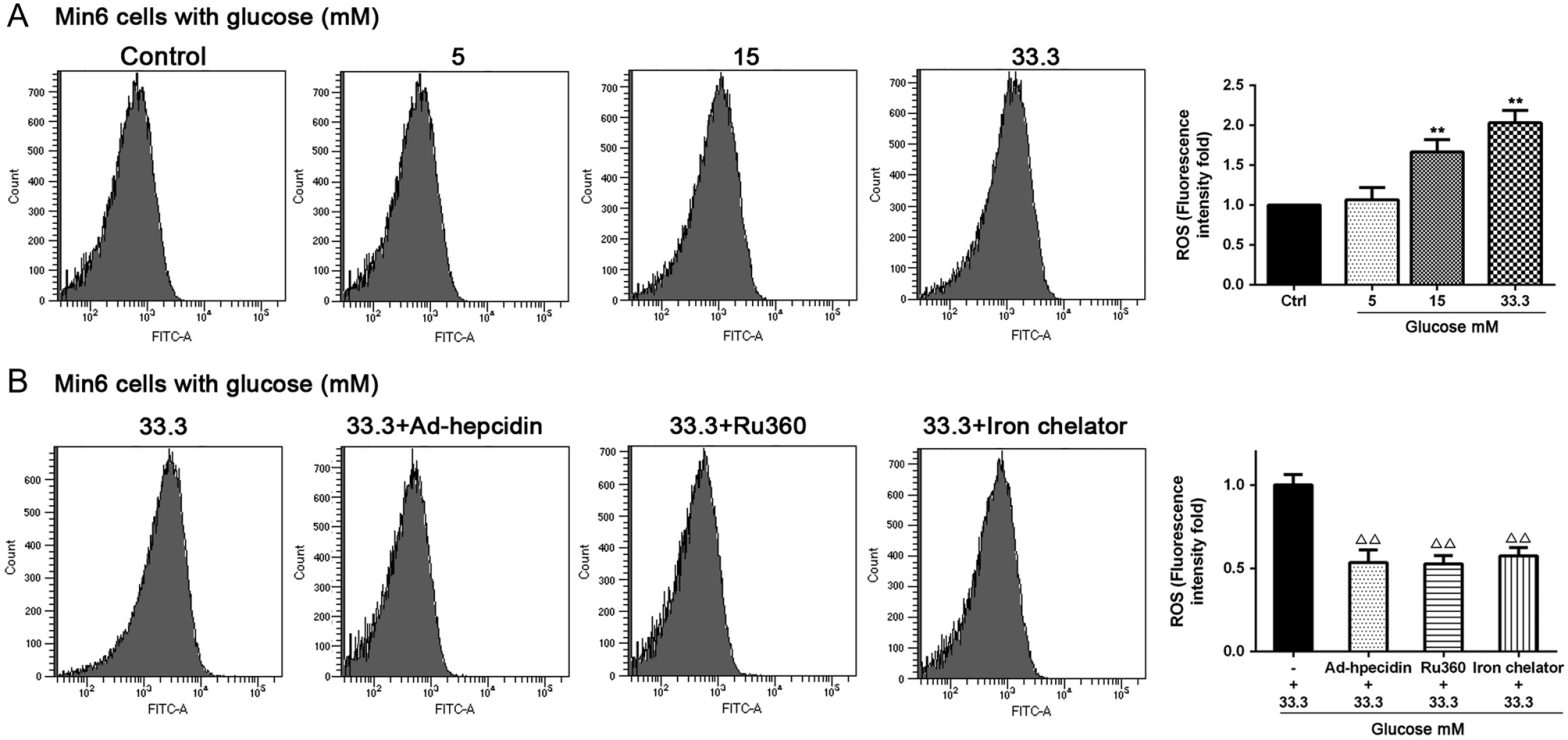
The level of ROS was measured by DCFH-DA staining and flow cytometry analysis following treatment with different concentrations of glucose for 48 h in Min6 cells. Statistical graph of DCFH-DA green fluorescence-positive cells as the fold change compared to the control. **indicate *P* < 0.01 compared with the control group (A). Min6 cells were infected with Ad-hepcidin or treated with RU 360 or an iron chelator plus 33.3 mM glucose. The level of ROS was measured via DCFH-DA staining and flow cytometry. ^△△^indicate *P* < 0.01 compared with the 33.3 mM glucose group (B).

The excess iron in the cytoplasm could be transported into mitochondria via the mitochondrial uniporter (Mcfu). The effect of iron toxicity on mitochondria is associated with the mitochondrial production of OH**^·^**·radicals according to the Fenton reaction mechanism (Fe^2^ + H_2_O_2_→Fe^3+^ + (OH)^−^ + OH**^·^**) (29). As expected, hyperglycemia increased the level of ROS in Min6 cells (Fig. 3A). To determine if this increase in ROS was mediated by an iron overload, we pretreated cells with an iron scavenger (iron chelator), Mcfu inhibitor (Ru360) or infected Ad-hepcidin virus and subjected the cells to high glucose stimulation. The ROS content decreased in all groups compared with the 33.3 mM glucose treatment group (Fig. 3B). There was no statistically significant difference in ROS content between the 33.3 mM group and the 33.3 mM + Ad-Gfp group (*P* = 0.38), nor was there any statistically significant difference between the control, Ad-hepcidin, Ru 360 and iron chelator groups (Supplementary Fig. 3).

### Excess mitochondrial iron induces ΔΨm depolarization and inhibits ATP synthesis

**Figure 4:**
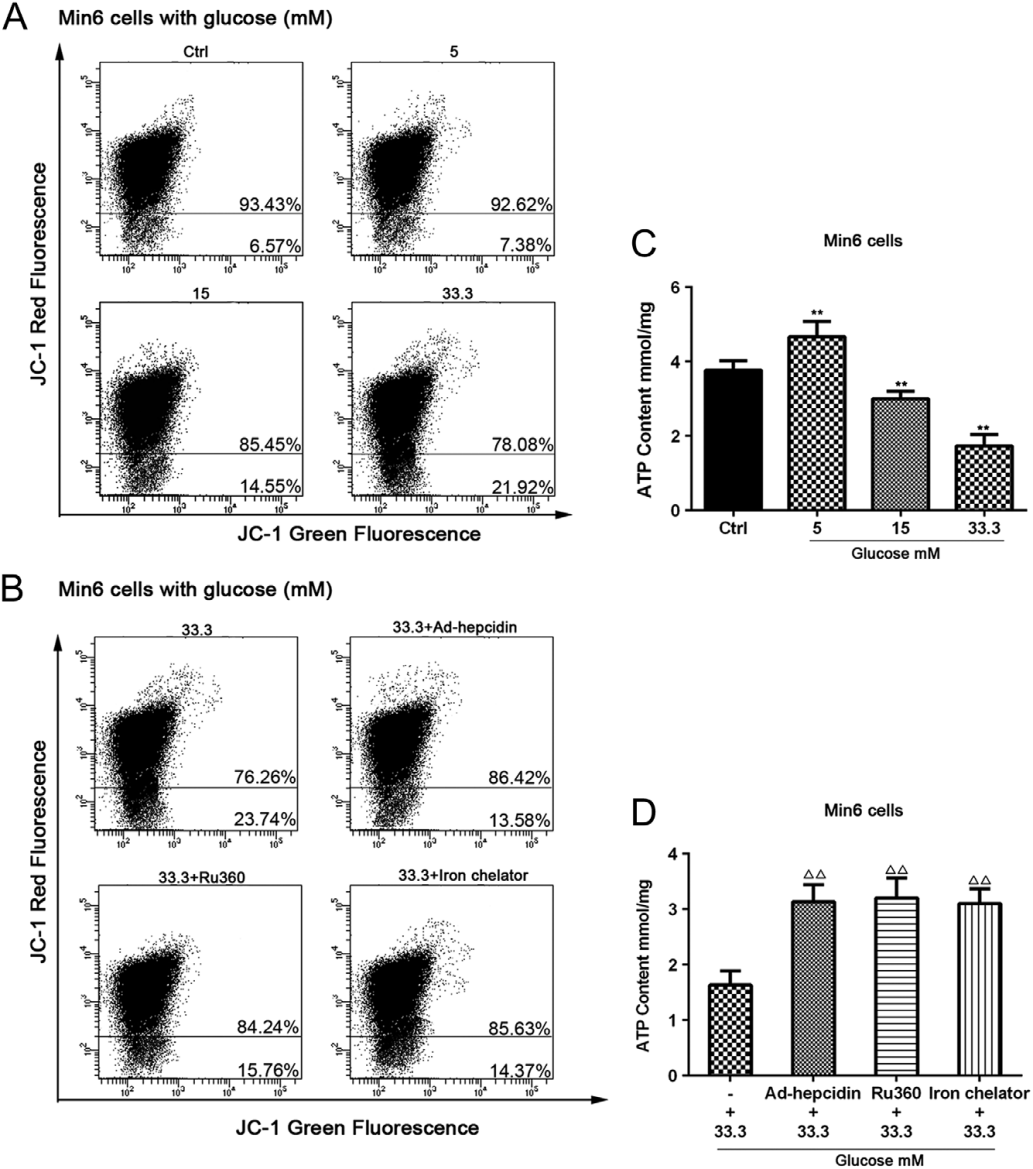
The ΔΨm was measured by JC-1 staining and flow cytometry analysis following treatment with different glucose concentrations for 48 h in Min6 cells. Statistical percentage of JC-1 red fluorescence and green fluorescence is shown in picture (A). Min6 cells were infected with Ad-hepcidin or treated with RU 360 or iron chelator plus 33.3 mM glucose. The ΔΨm was measured by JC-1 staining and flow cytometry analysis (B). The ATP content was measured by ATP fluorescence analysis following treatment with various glucose concentrations for 48 h in Min6 cells. ** indicate *P* < 0.01 compared with the control group (C). Min6 cells were infected with Ad-hepcidin or treated with RU 360 or iron chelator plus 33.3 mM glucose and the level of ATP was measured. ^△△^ indicate *P* < 0.01 compared with the 33.3 mM glucose group (D).

Excess Fe^2+^ transported into mitochondria causes inner membrane depolarization which inhibited oxidative respiratory chain and electron transfer, and ATP generation is depressed. The mitochondrial membrane potential was analyzed by JC-1 staining. The results indicate an obvious disruption of the mitochondrial membrane potential in Min6 cells under hyperglycemia stimulation (Fig. 4A). The iron content in cytoplasm was corrected by pretreating cells with an iron chelator, Ru360 and infected Ad-hepcidin virus. The ratio of red fluorescence/green fluorescence was increased compared with the hyperglycemia stimulation group (Fig. 4B). Similarly, when the ATP content was observed, hyperglycemia decreased ATP content (Fig. 4C), whereas treatment with the iron chelator, Ru360, and overexpression of hepcidin could recover the level of ATP (Fig. 4D). There was no statistically significant difference in the ratio of red fluorescence/green fluorescence and ATP content between the 33.3 mM group and the 33.3 mM + Ad-Gfp group (*P* = 0.28, *P* = 0.37), nor was there any statistically significant difference between the control, Ad-hepcidin, Ru 360 and iron chelator groups (Supplementary Fig. 4).

### Effects of iron restriction on blood glucose levels and insulin levels in *db/db* mice

**Figure 5:**
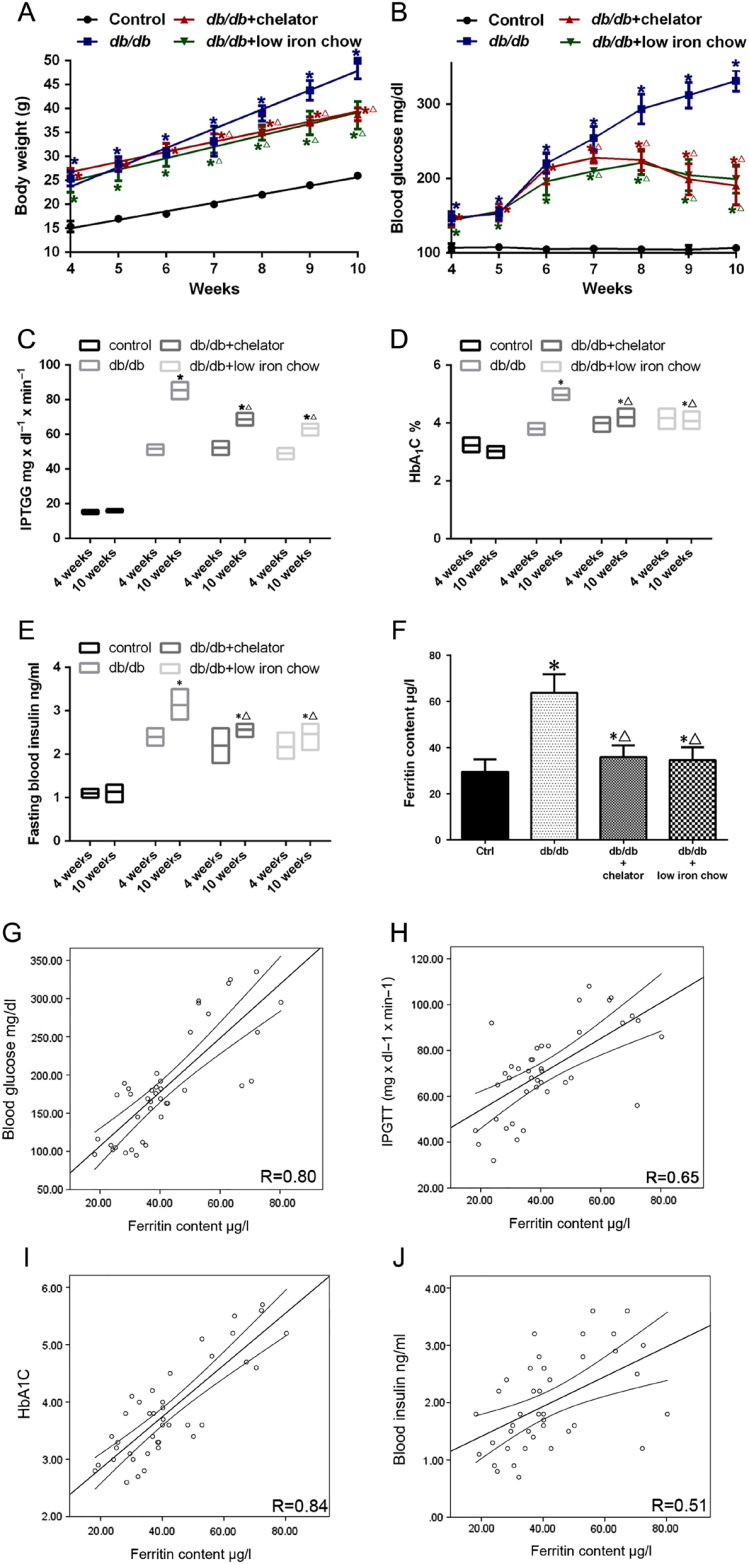
*db/db* mice were fed either a normal diet chow, low iron content diet chow or iron chelator for 7 weeks. Body weight and fasting blood glucose (FBG) levels were recorded every week and then integrated as curve chart (A and B). IPGTT, HbA_1_C% and fasting insulin content (FIns) were recorded at 4 and 10 weeks (C, D, and E). The level of ferritin was measured using an ELISA (F). *indicates *P* < 0.05 compared with the control group. **^△^**indicates *P* < 0.05 compared with the *db/db* group. Correlations between ferritin levels and FBG, IPGTT, HbA_1_C%, FIns (G, H, I and J). Regression lines and 95% confidence intervals are plotted if statistical significance is present. *R* presented the correlation coefficient of linear regression models.

The results presented in the current manuscript suggest that lower hepcidin expression eventually leads to decreased insulin release (Fig. 1). In this case, we restricted the iron content of *db/db* mice by feeding the animals low iron chow or normal chow + iron chelator. An analysis of body weight, FBG, IPGTT, HbA_1_C% and FIns were performed. At 4 weeks, the FBG of *db/db* mice just started to raise compared with control group (Fig. 5B). There is no differences between the *db/db* animals under different treatment conditions (4 weeks).The mice on the low iron chow and iron chelator gained less weight than the *db/db* mice but remained significantly obese compared to the control mice (*P* < 0.01, Fig. 5A). Since body weight is a major determinant of the glucose tolerance status, we next studied whether these differences in weight might improve glucose tolerance. In the iron chelator and low iron chow groups, the IPGTT was higher compared with control group but was much improved compared to the *db/db* group, which is consistent with the relationship with body weight (Fig. 5C). We also observed a partial reversion on FBG, HbA1C% and FIns in the iron chelator and low iron chow groups when compared to control db/db mice (Fig. 5C, D and E). There was no difference of FBG, IPGTT, HbA_1_C% and FIns in control mice with normal chow or low iron chow or iron chelator except of body weight at 10 weeks (Supplementary Fig. 5). Iron restriction was associated with a considerable benefit in the blood glucose control. We adopted ferritin to judge the level of serum iron content in the mice. In the iron chelator and low iron chow groups, the ferritin level was slightly higher compared with the control group but was much improved compared to the *db/db* group (Fig. 5F). The linear regression models to reveal the relationship between iron content and FBG, IPGTT, HbA_1_C% and FIns. As expected, the higher of ferritin level, the worse of blood glucose level of mice (Fig. 5G, H, I and J).

## Discussion

**Figure 6:**
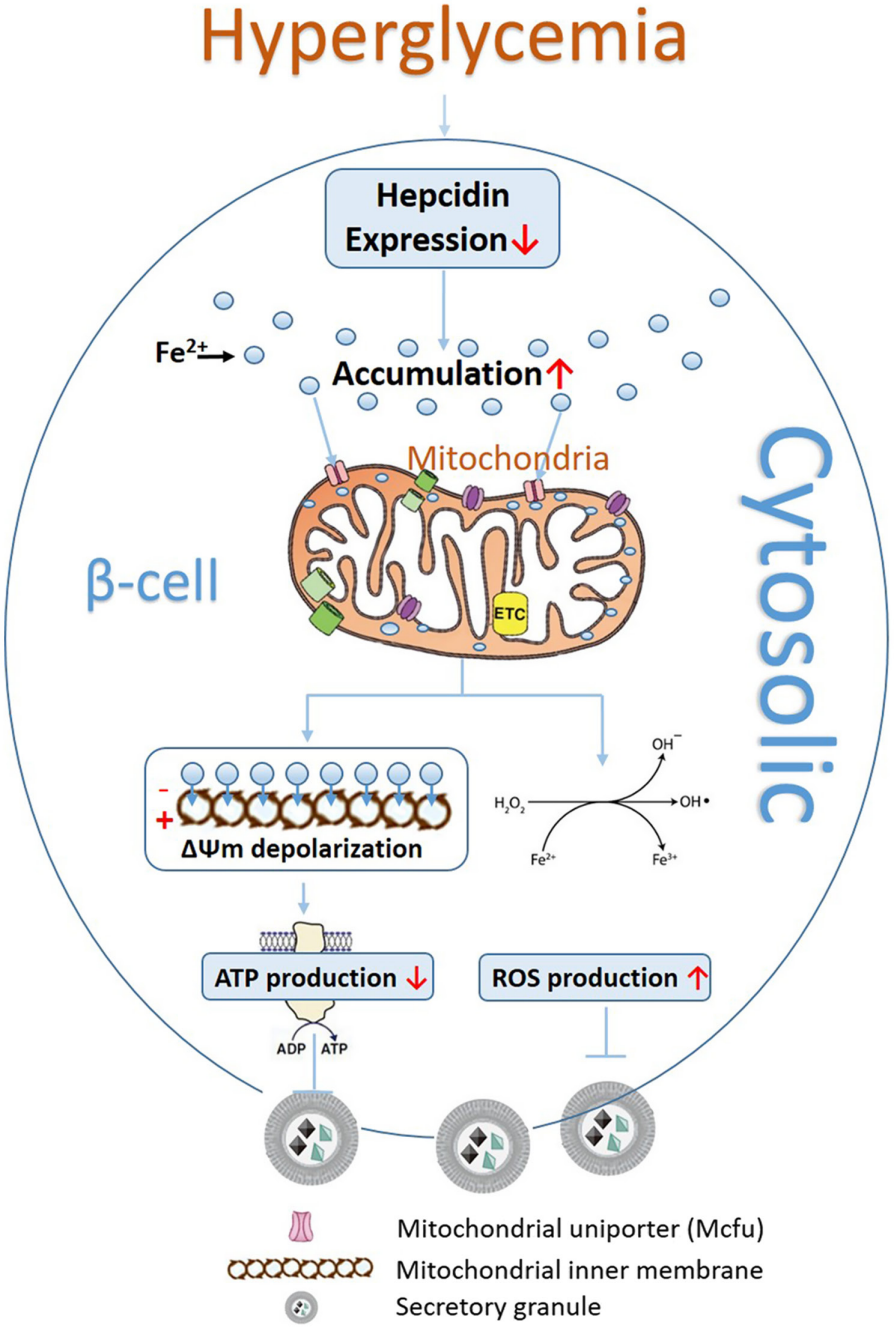
Diagram depicting the role of hepcidin-mediated glucose toxicity on pancreatic β cell. With hyperglycemia stimulation, hepcidin expression decreased leading to iron overload in the cytosol. The excessive iron could squeeze into mitochondria via Mcfu transported causing ΔΨm depolarization; inhibited ATP production and induced massive ROS production. The dysfunction of mitochondria inevitably led to insulin secretion decrease in pancreatic β cells.

Our previous study demonstrated that in addition to its role on iron regulation, hepcidin is involved in glucotoxicity-mediated impairment of pancreatic β-cell function by inhibiting insulin synthesis (14). Under hyperglycemic conditions, the expression of hepcidin was inhibited and the iron metabolism balance was disrupted. However, whether this iron metabolism disorder is related to the failure of β-cell function remains unknown. In the present study, we clearly demonstrated that a low expression of hepcidin leads to an iron overload in β-cells, a portion of excess Fe^2+^ that accumulates in the cytoplasm is stored in a stable complex as ferritin (2, 5), whereas the other portion is pumped into the mitochondria via Mcfu (17, 30). In the mitochondria, the accumulation of Fe^2+^ can impair mitochondrial function through: (1) the generation of a large amount of ROS via the Fenton/Haber–Weiss mechanism (Fe^2+^ + H_2_O_2_ → Fe^3+^ + (OH)^−^ + OH^•^) (18, 29); and (2) ΔΨm depolarization, which affects electron transport, damaging the mitochondrial aerobic respiratory pathway, and inhibiting ATP synthesis (31, 32). All of these toxic effects could be reversed by Ad-hepcidin infected, Ru360, and an iron chelator. GSIS function was also improved with a recovery in hepcidin levels. Our data clearly indicate that iron overload plays an important role in the mitochondria dysfunction mediated β-cell function under conditions of hyperglycemia (Fig. 6).

The regulation of hepcidin on iron metabolism mainly through iron absorption and iron exported pathway and was different in each tissue. Hepcidin-knockout mice develop iron overload in the liver and pancreas, but iron deficit in the macrophage-rich spleen (33). There should be a negative correlation between the iron content and ferroportin (FPN) expression level. However, we didn’t observe the high expression of FPN associated with low hepcidin expression in Min6 cells with 33.3 mM glucose stimulated 48 h (data not shown). We presumed the mechanism that hepcidin internalized FPN leading to its degradation clarified in other cell types was not conserved in pancreatic beta cell. There were several evidence supported our presumption. First FPN exported iron primarily from duodenal enterocytes, reticuloendothelial and macrophages. Although FPN was the main receptor for hepcidin and exercising the duty to iron exported, the mechanism of its action to hepcidin in other cells was not clearly understood (1). Second, there was not a connection between iron overload and FPN up-expression but with increased iron intake in Hansen’s work (34) which also confirmed by our study with high glucose stimulation, the DMT and TfR1 protein expression was increased (as Fig. 2C showed). This results indicated iron intake may involve in this process, but the specific molecular was not understand now.

Our results indicate that decreased iron deposition in the pancreatic tissue can decrease blood glucose in a T2DM animal and cell model, for which the associated mechanism has also been discussed above. Another study conducted by Cooksey also confirmed our resulted that restriction iron intake or with iron chelation could significantly ameliorate high blood glucose in *ob/ob* mice (27) (mouse model for type 2 diabetes, the* ob/ob Lep−/−*). But it showed a better effect on glucose control in iron chelator group than low iron chow diet group. While in our study the FBG, IPGTT, HbA_1_C% and FIns level between this two groups with no statistical difference in *db/db* mice. In Cooksey’t study, they inferred that 35 mg/kg iron content chow would induce iron-deficiency anemia in *ob/ob* mice which restricted hypoglycemic effect. In our study, with this dosage iron diet feed, the hemoglobin (Hb) content was maintained at normal levels at 10 week, once the diet contains less than 20 mg/kg iron, the mice began to displayed low levels of Hb, erythrocyte mean corpuscular volume (MCV), mean corpuscular hemoglobin (MCH), and were diagnosed as having iron-deficiency anemia at 10 weeks (Supplementary Fig. 6). We also found body weight was lower in iron restriction diet group (Supplementary Fig. 5). The reason for slower weight increase in mouse due to iron restriction was unknown yet.

Although we achieved a satisfactory improvement in blood glucose for the early onset of T2DM in *db/db* mice (10 weeks) with iron restriction, whether iron treatment will have an effect in a long-term group of *db/db* mice remains unknown. In the early stage of T2DM, the effect of impaired ROS production on mitochondrial function and the impact of oxidative stress on β-cell function are reversible. Thus, alleviating the toxic effects of iron accumulation could significantly restore the function of β-cells. However, when β-cell functionality has been irreparable destroyed, simply correcting the expression of hepcidin and inhibiting iron deposition may have little to no effect on blood glucose levels. At this stage, improving insulin signaling and insulin resistance would be a more appropriate treatment (40).

In conclusion, we have identified a hepcidin-mediated pathway of glucotoxicity, resulting in impaired β-cell functionality. The decreased hepcidin expression could lead to iron accumulation in the cytoplasm and mitochondria. The mitochondrial membrane potential was depolarized, which inhibited ATP synthesis and promoted excessive ROS production inducing oxidative stress. Abnormal iron metabolism in the mitochondria eventually impaired insulin secretion as Fig. 6 showed. Relieved iron overload status had a positive effect on the blood glucose control in the early onset of T2DM in *db/db* mice. Thus, our study may reveal the mechanism involved in the role of hepcidin in the glucotoxcity impaired pancreatic β cell function pathway.

## Supplementary Material

Supporting Figure 1

Supporting Figure 2

Supporting Figure 3

Supporting Figure 4

Supporting Figure 5

Supporting Figure 6

Supporting Table 1

Supporting Table 2

## Declaration of interest

The authors declare that there is no conflict of interest that could be perceived as prejudicing the impartiality of the research reported.

## Funding

This work was supported by a grant from the Health and Family Planning Commission of Wuxi City (Q201739).

## Author contribution statement

All authors took part in the conception and design of the study, as well as either drafting or critically revising the manuscript. All authors have approved the final version of the manuscript. Tingting Shu, Zhigang Lv, Yuchun Xie and Junming Tang collected the data and carried out the data analysis. Xvhua Mao is responsible for the integrity of the work as a whole.

## Data availability statement

The datasets used and analyzed during the current study are available from the corresponding author on reasonable request.
