# Supplementary figures and images for: Hepcidin as a key iron regulator mediates glucotoxicity-induced pancreatic β-cell dysfunction
Source: Endocr Connect. 2019 Jan 21;8(3):150–61. doi: 10.1530/EC-18-0516 (PMC6391907; doi:10.1530/EC-18-0516)

## Sp Fig. 1

Images of isolated islets stained for nuclei (DAPI, Blue) and insulin (Green).

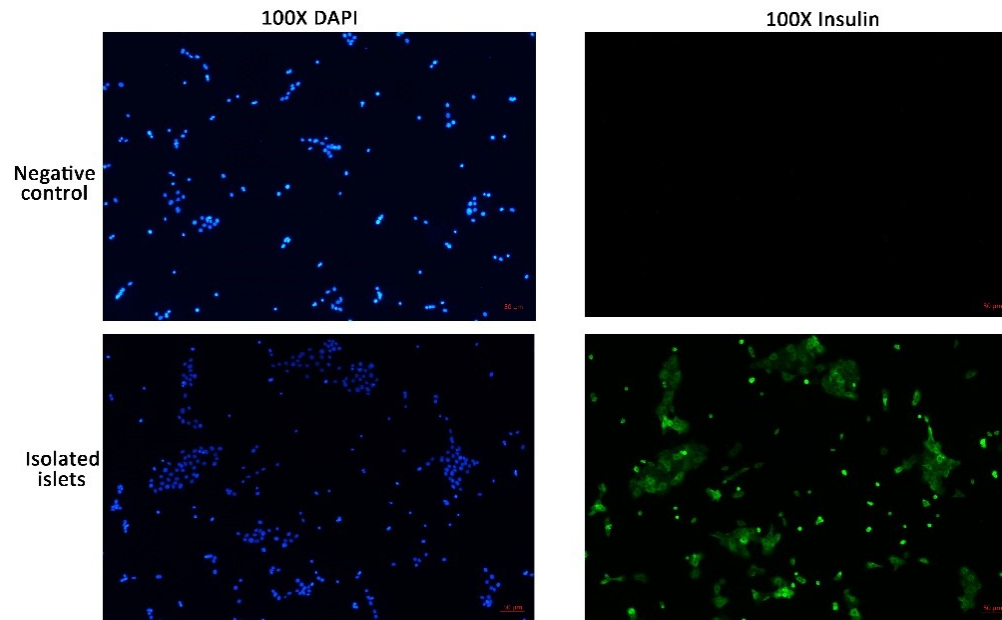

Supplement: Supporting Figure 1 [file supplementary_figure_1.pdf]
