# Supplementary material for: Hepcidin as a key iron regulator mediates glucotoxicity-induced pancreatic β-cell dysfunction
Source: Endocr Connect. 2019 Jan 21;8(3):150–61. doi: 10.1530/EC-18-0516 (PMC6391907; doi:10.1530/EC-18-0516)

Sp Fig. 2

Hepcidin was overexpressed in Min6 cells. Min6 cells were infected with adenovirus (Ad-*hepcidin*) or control adenovirus (Ad-*Gfp*,) for 24 h, after which the *hepcidin* mRNA expression was measured. ** indicates $P < 0.01$ compared with the Ad-Gfp group.

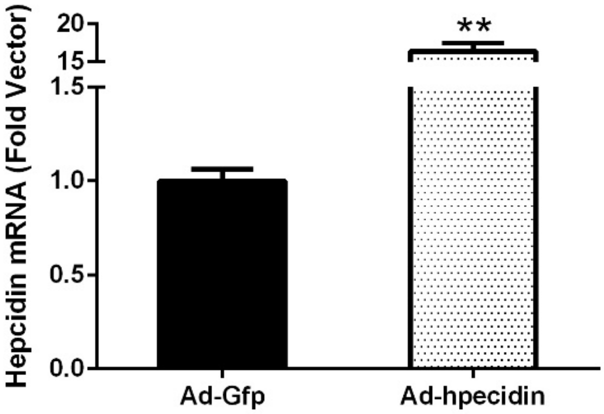

Supplement: Supporting Figure 2 [file supplementary_figure_2.pdf]
