# Supplementary material for: Hepcidin as a key iron regulator mediates glucotoxicity-induced pancreatic β-cell dysfunction
Source: Endocr Connect. 2019 Jan 21;8(3):150–61. doi: 10.1530/EC-18-0516 (PMC6391907; doi:10.1530/EC-18-0516)

Sp Fig. 4

Min6 cells were infected with Ad-hepcidin or treated with Ru 360 or an iron chelator for 48 h. Following JC-1 staining, the ratio of red fluorescence/green fluorescence (A) and ATP content (B) was determined.

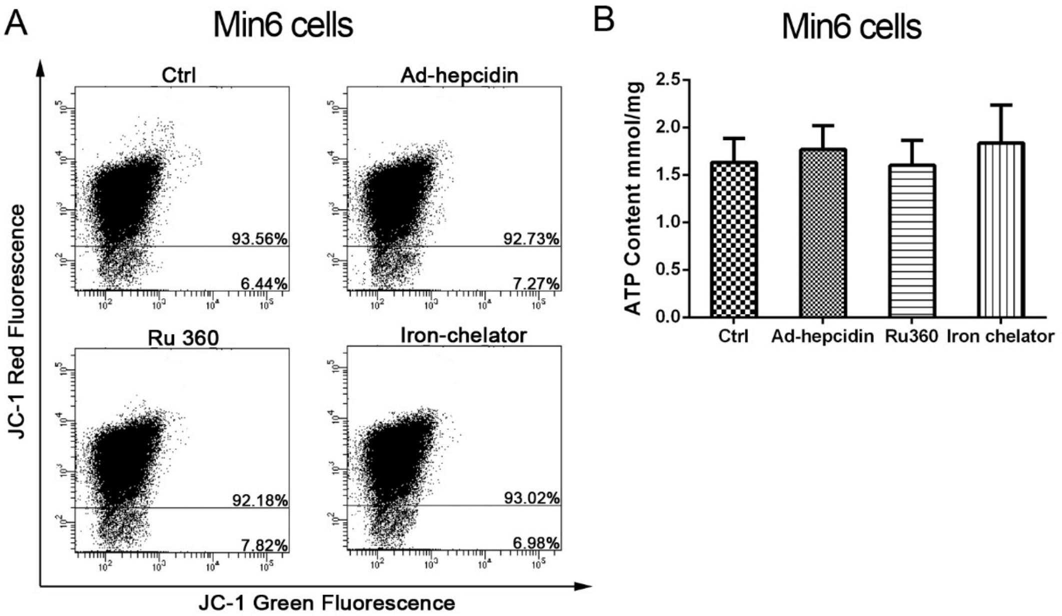

Supplement: Supporting Figure 4 [file supplementary_figure_4.pdf]
