# Supplementary material for: Hepcidin as a key iron regulator mediates glucotoxicity-induced pancreatic β-cell dysfunction
Source: Endocr Connect. 2019 Jan 21;8(3):150–61. doi: 10.1530/EC-18-0516 (PMC6391907; doi:10.1530/EC-18-0516)

Sp Fig. 5

Control mice were fed either a diet of normal chow, low iron content chow, or normal diet chow + iron chelator for seven weeks. Body weight, fasting blood glucose (FBG) levels, IPGTT, $HbA_1C\%$ and fasting insulin content (FIns), and ferritin levels were recorded at 4 and 10 weeks (A to F). *indicates $P < 0.05$ compared with the control group.

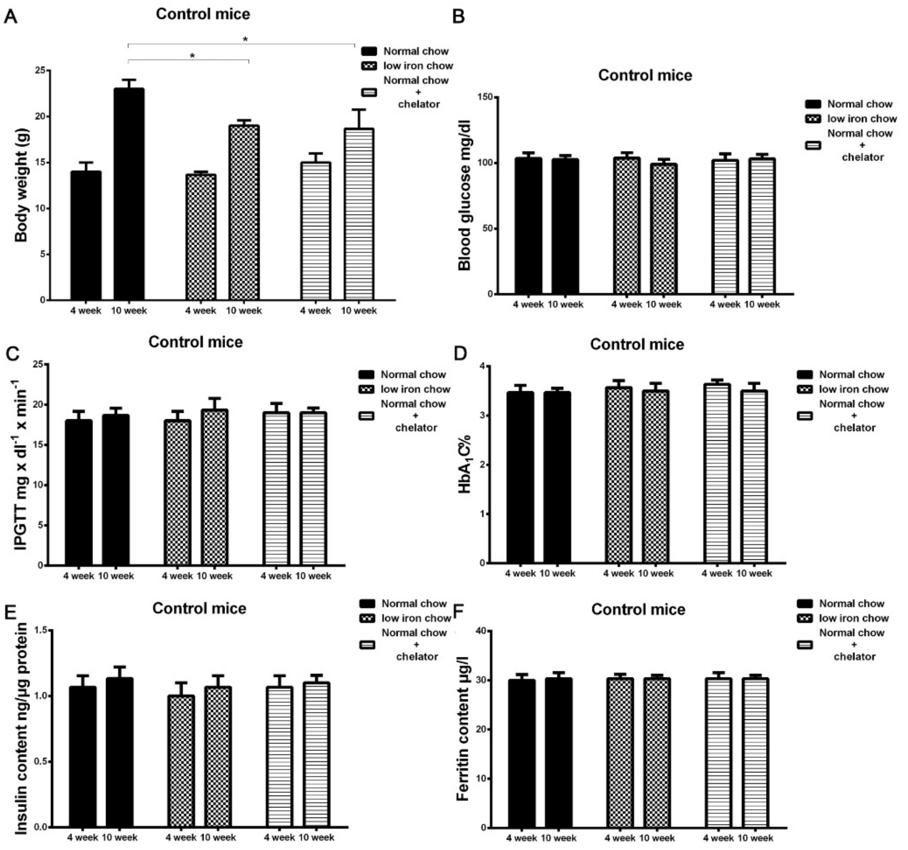

Supplement: Supporting Figure 5 [file supplementary_figure_5.pdf]
