# Supplementary material for: Hepcidin as a key iron regulator mediates glucotoxicity-induced pancreatic β-cell dysfunction
Source: Endocr Connect. 2019 Jan 21;8(3):150–61. doi: 10.1530/EC-18-0516 (PMC6391907; doi:10.1530/EC-18-0516)

Sp Fig6

Hemoglobin (Hb) level was determined in control and *db/db* mice with normal diet or with iron chelator +normal diet or with low iron diet (35 mg/kg) at 10 week (A). Hb level was determined in control and db/db mice with normal diet or with low iron diet (20 mg/kg) at 10 week (B). *indicates P < 0.05 compared with the normal diet in control group. △indicate *P* < 0.01 compared with compared with the normal diet in *db/db* group (B).

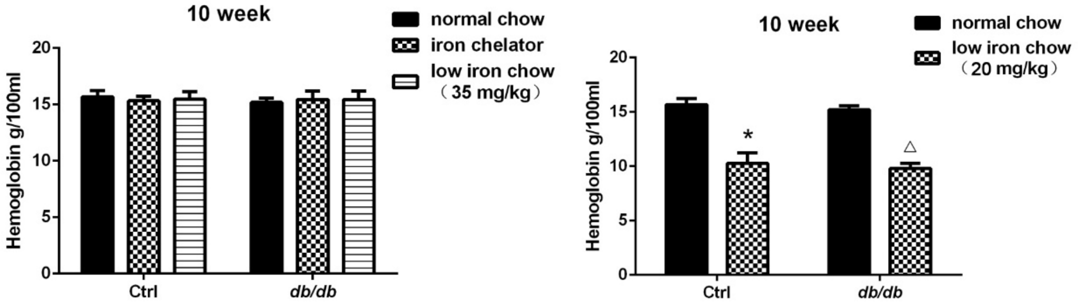

Supplement: Supporting Figure 6 [file supplementary_figure_6.pdf]
