# Supplementary material for: Hepcidin as a key iron regulator mediates glucotoxicity-induced pancreatic β-cell dysfunction
Source: Endocr Connect. 2019 Jan 21;8(3):150–61. doi: 10.1530/EC-18-0516 (PMC6391907; doi:10.1530/EC-18-0516)
Supplement: Supporting Table 1 [file supplementary_table_1.pdf]

Table1

qRT-RCR primers used for *hepcidin* mRNA level detection

Mouse *hepcidin* primers:	Forward: 5'-AAGCTTATGCCTTAGACTGCACA-3'<br>Reverse: 5'-ATGAAGACGATTTTATTTTCAGAATTC-3'
Mouse *β-actin* primers:	Forward: 5'-CAAGGCCAACCGTGAAAAGAT-3'<br>Reverse: 5'-AATGCCAGTGGTACGACCAGAG-3'
