# Supplementary material for: Hepcidin as a key iron regulator mediates glucotoxicity-induced pancreatic β-cell dysfunction
Source: Endocr Connect. 2019 Jan 21;8(3):150–61. doi: 10.1530/EC-18-0516 (PMC6391907; doi:10.1530/EC-18-0516)
Supplement: Supporting Table 2 [file supplementary_table_2.pdf]

Table2

Fish probes used for *hepcidin* mRNA expression in mouse pancreatic tissue

Mouse *Hamp*	5'-*FAM*- UGCAACAGAUACCACACUGGGAAUUGUUAC -3'
Mouse *Ins2*	*Ins2* 5'-Cy3- CACAGGGCCAUGUUGAAACAAUAACCUUCC-3'
